# Association of Dyslipidemia with Renal Outcomes in Chronic Kidney Disease

**DOI:** 10.1371/journal.pone.0055643

**Published:** 2013-02-04

**Authors:** Szu-Chia Chen, Chi-Chih Hung, Mei-Chuan Kuo, Jia-Jung Lee, Yi-Wen Chiu, Jer-Ming Chang, Shang-Jyh Hwang, Hung-Chun Chen

**Affiliations:** 1 Division of Nephrology, Kaohsiung Medical University Hospital, Kaohsiung Medical University, Kaohsiung, Taiwan; 2 Department of Internal Medicine, Kaohsiung Municipal Hsiao-Kang Hospital, Kaohsiung Medical University, Kaohsiung, Taiwan; 3 Faculty of Renal Care, College of Medicine, Kaohsiung Medical University, Kaohsiung, Taiwan; 4 Faculty of Medicine, College of Medicine, Kaohsiung Medical University, Kaohsiung, Taiwan; University of Sao Paulo Medical School, Brazil

## Abstract

Dyslipidemia is highly prevalent in patients with chronic kidney disease (CKD) and the relationship between dyslipidemia with renal outcomes in patients with moderate to advanced CKD remains controversial. Hence, our objective is to determine whether dyslipidemia is independently associated with rapid renal progression and progression to renal replacement therapy (RRT) in CKD patients. The study analyzed the association between lipid profile, RRT, and rapid renal progression (estimated glomerular filtration rate [eGFR] slope <−6 ml/min/1.73 m^2^/yr) in 3303 patients with stages 3 to 5 CKD. During a median 2.8-year follow-up, 1080 (32.3%) participants commenced RRT and 841 (25.5%) had rapid renal progression. In the adjusted models, the lowest quintile (hazard ratios [HR], 1.23; 95% confidence interval [CI], 1.01 to 1.49) and the highest two quintiles of total cholesterol (HR, 1.25; 95% CI, 1.02 to 1.52 and HR, 1.35; 95% CI, 1.11 to 1.65 respectively) increased risks for RRT (*vs.* quintile 2). Besides, the highest quintile of total cholesterol was independently associated with rapid renal progression (odds ratio, 1.36; 95% CI, 1.01 to 1.83). Our study demonstrated that certain levels of dyslipidemia were independently associated with RRT and rapid renal progression in CKD stage 3–5. Assessment of lipid profile may help identify high risk groups with adverse renal outcomes.

## Introduction

Chronic kidney disease (CKD) results in profound dysregulation of several key enzymes and metabolic pathways that eventually contributes to disordered high-density lipoprotein (HDL) cholesterol and triglyceride-rich lipoproteins [1]. With the progression of CKD, these metabolic derangements may be further worsened and participate in atherogenic diathesis and possibly renal functional progression itself [2]. A large number of epidemiologic studies have suggested the independent role of dyslipidemia on cardiovascular morbidity and mortality in the general population [3], [4]. In CKD populations, the relationship of dyslipidemia with cardiovascular disease is inconclusive and paradoxical [5]. However, published data regarding the relationship between dyslipidemia and renal outcomes in moderate to advanced CKD stages are limited.

Previous animal studies have shown a correlation between the presence of an atherogenic lipid profile and the onset of glomerulosclerosis and endothelial dysfunction [6]–[9]. Consistent with the experimental model, dyslipidemia in humans might be associated with development and progression of renal dysfunction [10], [11]. Among human studies relating dyslipidemia to renal outcome, one study found that higher total cholesterol, higher non-HDL-cholesterol and lower HDL-cholesterol were significantly associated with an increased risk of developing renal dysfunction in healthy men [10]; one study suggested a weak association in type 1 diabetes mellitus (DM) [11]; another study disclaimed this association in non-diabetic patients with stage 3 to 4 CKD [12]. Data concerning this effect on kidney disease progression in patients with mild to moderate kidney failure are also conflicting [10]–[12]. A recent large randomized control trial showed that statins treatment lowered low-density lipoprotein (LDL) cholesterol, but had no substantial effect on kidney disease progression in patients with CKD [13]. Thus, the role of dyslipidemia as an independent risk marker for adverse renal outcomes still remains uncertain in moderate to advanced CKD.

In the present study, we investigated 3,303 patients in stages 3 to 5 CKD from a hospital-based CKD care system in southern Taiwan to assess whether dyslipidemia was independently associated with renal replacement therapy (RRT) and rapid renal progression.

## Materials and Methods

### Participants and Measurements

Between November 11, 2002 and May 31, 2009, 3749 patients who joined the ICKD (Integrated CKD care program Kaohsiung for delaying dialysis) prospective observation study from two affiliated hospitals (one medical center and another regional hospital) of Kaohsiung Medical University were included and followed until May 31, 2010. The definition of CKD followed the National Kidney Foundation-Kidney Disease Outcomes Quality Initiative (K/DOQI) guidelines and the CKD stage was defined by using patients' baseline estimated glomerular filtration rate (eGFR) [14]. Their baseline renal functions were estimated by using the average of two eGFR values determined three months before and three months after enrollment. Most of the participants were referred from primary care physicians or from doctors of non-nephrology specialties in the two hospitals for their impairment or progression of renal function. Ninety patients were lost to follow-up in less than 3 months. Three hundred and fifty-six patients were CKD stages 1 and 2, and the final study population consisted of 3303 patients with CKD stages 3 to 5.

Baseline variables included demographic features, medical history (DM, hypertension and cardiovascular disease), body mass index (BMI), mean arterial pressure, laboratory data (albumin, hemoglobin, triglyceride, total cholesterol, HDL-cholesterol, LDL-cholesterol, non-HDL-cholesterol, C-reactive protein (CRP), glycated hemoglobin (HbA1c), uric acid, total calcium, phosphate and urine protein-to-creatinine ratio), and medication history (statins and fibrates). The demographic features were the baseline record and the medical history was obtained by medical chart review. Mean arterial pressure was calculated by the averaged systolic and diastolic blood pressure measured three months before and after enrollment. The laboratory data three months before and after enrollment of the CKD care system were averaged and analyzed. The condition of treatment (used or not used) with statins or fibrates was collected at the beginning.

### Ethics Statement

The study protocol was approved by the Institutional Review Board of the Kaohsiung Medical University Hospital (KMUH-IRB-20120232). Informed consents were obtained in written form from patients and all clinical investigation was conducted according to the principles expressed in the Declaration of Helsinki. The patients gave consent for the publication of the clinical details.

### Quantification of renal function and progression

Kidney function was examined by using eGFR derived from the simplified Modification of Diet in Renal Disease (MDRD) Study equation. The equation was eGFR ml/min/1.73 m^2 = ^186×Serum creatinine ^−1.154^×Age^−0.203^×0.742 (if female) [15]. The rate in renal function decline was assessed by the eGFR slope, defined as the regression coefficient between eGFR and time in unit of ml/min/1.73 m^2^/year. Two renal outcomes were accessed: RRT and rapid renal progression. The RRT was ascertained by reviewing medical charts or catastrophic illness certificates (issued by the Bureau of National Health Insurance, Taiwan) and defined as patients needing the commencement of hemodialysis, peritoneal dialysis, or renal transplantation. The timing for RRT was regulated by the Bureau of National Health Insurance regarding the laboratory data, nutritional status, uremic symptoms, and creatinine clearance. Rapid renal progression was defined as the lowest quartile (the eGFR slope <−6 ml/min/1.73 m^2^/yr, an integer near the cut point between the lowest two quartiles of the eGFR slope). Models for RRT were censored at the commencement of RRT, death, or at the end of the follow-up.

### Statistical analysis

Summary statistic results of baseline characteristics of all subjects and stratification by quintiles of total cholesterol are expressed as percentages for categorical data, mean ± standard deviation for continuous variables with approximately-normal distribution, and median and interquartile range for continuous variables with skewed distribution.

Cox proportional hazards analysis was used for evaluating the relationship between quintiles of lipid profile and RRT. Multiple logistic regression analyses were used to evaluate the relationship between quintiles of lipid profile and rapid renal progression. The cutoff values of quintiles of total cholesterol were <155, 155–179, 180–200, 201–229, and ≥230 mg/dL respectively. Quintile 2 of lipid profile, i.e. total cholesterol, HDL-cholesterol, LDL-cholesterol, non-HDL-cholesterol, and triglyceride, were taken as reference category, which was the lowest risk group for the outcomes. Covariates were included into these models if their *P* value was less than 0.05 in univariate analysis and skewed distributed continuous variables were log-transformed to attain normal distribution. The adjusted covariates included age, sex, DM, cardiovascular disease, current smoker, BMI, mean arterial pressure, eGFR, log-transformed urine protein, albumin, hemoglobin, log-transformed CRP, HbA1c, uric acid, phosphate, statins and fibrates use.

## Results

Clinical characteristics of patients with different cholesterol quintiles are shown in Table 1. A total of 3303 non-dialyzed CKD patients were included. The mean age was 63.5±13.5 years and there were 1909 males and 1394 females. The mean eGFR and total cholesterol level were 24.7±15.1 ml/min/1.73 m^2^ and 195.7±53.7 mg/dl. There were 32.7% and 8.3% of study subjects treated with statins and fibrates at baseline respectively. The underlying etiology of CKD included 1258 with diabetic kidney disease (38.1%), 1168 with chronic glomerular diseases (35.4%), 300 with tubulointerstitial diseases (9.1%), 368 cases caused by hypertension (11.1%), and 208 caused by other diseases (6.3%). Numbers of patients in different CKD stages were approximately even: 35.8% in stage 3, 29.1% in stage 4, and 35.1% in stage 5. Compared with patients with quintile 2, patients with the lowest quintile had high prevalence of albumin <3.5 g/dL (28.1% *versus* 22.0%), and higher CRP.

**Table 1:** Baseline Characteristics of the 3303 CKD Stage 3–5 Subjects by total cholesterol quintiles.

Variable	All subjects (n = 3303)	Quintile 1 (<155 mg/dL) (n = 683)	Quintile 2 (155–179 mg/dL) (n = 660)	Quintile 3 (180–200 mg/dL) (n = 689)	Quintile 4 (201–229 mg/dL) (n = 643)	Quintile 5 (≥230 mg/dL) (n = 628)	*P* for trend
Total cholesterol (mg/dL)	195.7±53.7	134.3±17.2	168.8±7.1	191.8±6.3	215.9±8.2	274.6±56.4	< 0.001
HDL-cholesterol (mg/dL)	42.4±13.8	35.7±11.0	40.5±11.8	43.5±13.6	44.7±14.2	48.2±15.0	< 0.001
LDL-cholesterol (mg/dL)	112.9±38.8	77.1±22.3	98.1±23.8	113.1±21.1	126.9±24.9	153.0±47.0	< 0.001
Non-HDL cholesterol (mg/dL)	153.3±51.6	98.6±19.0	128.3±13.5	148.2±14.6	171.2±16.3	226.4±57.6	< 0.001
Triglyceride (mg/dL)	126.5 (91–184)	100 (73–142)	115 (84.4–160)	121.5 (92–172.9)	137 (102–191.4)	179 (123–255)	< 0.001
Demographics and Medical History							
Age (years)	63.5±13.5	65.5±13.2	64.3±13.2	64.4±13.9	62.3±13.5	60.6±1.32	< 0.001
Gender (male%)	42.2	32.7	36.2	42.2	46.7	54.5	< 0.001
Hypertension (%)	67.1	66.6	70.0	66.0	66.4	66.6	0.532
Diabetes mellitus (%)	44.6	45.2	46.4	40.6	43.2	47.6	0.831
Cardiovascular disease (%)	26.4	30.6	28.0	25.4	26.1	21.7	< 0.001
Current smoker (%)	11.1	12.7	9.8	11.6	10.9	11.6	0.936
BMI (m^2^/kg)	24.7±4.0	24.3±4.0	24.8±3.9	24.8±3.9	24.8±3.8	25.0±4.3	0.005
MAP (mmHg)	100.0±13.8	97.3±13.8	98.5±12.9	100.1±14.2	101.0±13.6	103.3±13.5	< 0.001
Renal Function Status							
CKD stage							
Stage 3 (%)	35.8	31.6	36.1	39.5	36.5	39.5	0.004
Stage 4 (%)	29.1	26.2	28.5	29.5	28.6	29.5	
Stage 5 (%)	35.1	42.2	35.5	31.1	34.8	31.1	
eGFR (ml/min/1.73 m^2^)	24.7±15.1	22.8±14.9	24.8±15.0	26.1±15.1	24.9±15.4	25.0±14.9	0.015
Urine protein-to-creatinine ratio	1118.3 (408.6–2521.3)	1047.1 (408.7–2089.3)	1000 (370.4–2187.8)	958 (331–2033.6)	1122.3 (443.5–2477.1)	1864.5 (632.3–4243.2)	< 0.001
Laboratory Data							
Albumin (g/dL)	3.8±0.5	3.8±0.5	3.9±0.5	3.9±0.5	3.9±0.5	3.7±0.7	0.013
Hemoglobin (g/dL)	10.9±2.4	10.4±2.4	10.9±2.4	11.2±2.4	11.1±2.3	11.1±2.2	< 0.001
C-reactive protein (mg/L)	1.2 (0.4–5.4)	1.6 (0.5–8.0)	1.0 (0.4–5.0)	1.2 (0.4–5.0)	1.2 (0.4–5.6)	0.9 (0.4–4.0)	< 0.001
HbA1c (%)	6.5±1.6	6.2±1.5	6.4±1.4	6.4±1.5	6.5±1.5	6.9±2.0	< 0.001
Uric Acid (mg/dL)	7.9±2.0	8.0±1.9	7.9±2.1	7.9±2.0	7.9±1.9	7.9±1.9	0.452
Total Calcium (mg/dL)	9.1±0.8	9.0±0.8	9.1±0.8	9.1±0.7	9.1±0.8	9.1±0.8	0.024
Phosphate (mg/dL)	4.4±1.3	4.5±1.3	4.3±1.2	4.3±1.2	4.5±1.3	4.6±1.3	0.004
Medication Prescription							
Statin (%)	32.7	28.0	30.6	32.2	33.7	39.6	< 0.001
Fibrate (%)	8.3	8.8	6.2	8.3	9.3	8.9	0.335
Outcome							
Days of follow-up (days)	1150.3±577.6	1044.8±593.5	1166.9±560.1	1163.3±578.8	1217.0±574.4	1165.3±566.6	< 0.001
eGFR slope (ml/min/1.73 m^2^/yr)	−2.2 (−5.6 to −0.1)	−2.1 (−5.2 to −0.1)	−2.1 (−5.0 to 0.0)	−1.9 (−4.8 to 0.1)	−2.0 (−5.2 to −0.1)	−3.3 (−7.8 to −0.6)	< 0.001
Renal replacement therapy (%)	32.7	33.4	30.3	27.7	34.2	38.4	0.053
Rapid renal progression (%)	23.5	21.8	19.9	20.7	22.6	32.9	< 0.001

Data expressed as mean ± standard deviation, median (interquartile range) or percentage.

Abbreviations: HDL, high-density lipoprotein; LDL, low-density lipoprotein; BMI, body mass index; MAP, mean arterial pressure; CKD, chronic kidney disease; eGFR, estimated glomerular filtration rate; HbA1c, glycated hemoglobin.

### Total cholesterol quintiles and RRT

There were 1080 patients (32.7%) commencing RRT during a median approximately 2.8-year follow-up, including hemodialysis (n = 957), peritoneal dialysis (n = 116) and renal transplant (n = 7). Table 2 shows a Cox proportional hazards regression analysis for progression to RRT. In models adjusted for age, gender, DM, cardiovascular disease, current smoker, BMI, mean arterial pressure, eGFR, log-transformed urine protein, albumin, hemoglobin, log-transformed CRP, HbA1c, uric acid, phosphate, statins and fibrates use, the adjusted hazard ratios [HR] for quintile 1 *versus* quintile 2 was 1.23 (95% confidence interval [CI], 1.01 to 1.49, *P* = 0.037), for quintile 4 *versus* quintile 2 was 1.25 (95% CI, 1.02 to 1.52, *P* = 0.028), and for quintile 5 *versus* quintile 2 was 1.35 (95% CI, 1.11 to 1.65, *P* = 0.003). The lowest quintile and the highest two quintiles of total cholesterol increased risks for RRT.

**Table 2:** Total cholesterol and renal replacement therapy and rapid renal progression in all subjects by quintiles of total cholesterol.

	Renal replacement therapy		Rapid renal progression	
	Unadjusted	Adjusted	Unadjusted	Adjusted
total cholesterol	HR (95% CI)	HR (95% CI)	OR (95% CI)	OR (95% CI)
Quintile 1	1.33 (1.10, 1.61)*	1.23 (1.01, 1.49)*	1.12 (0.86, 1.46)	1.06 (0.79, 1.42)
Quintile 2	1	1	1	1
Quintile 3	0.90 (0.74, 1.10)	1.08 (0.88, 1.33)	1.05 (0.81, 1.38)	1.17 (0.87, 1.57)
Quintile 4	1.15 (0.95, 1.39)	1.25 (1.02, 1.52)*	1.18 (0.90, 1.54)	1.20 (0.89, 1.61)
Quintile 5	1.36 (1.13, 1.64)*	1.35 (1.11, 1.65)*	1.98 (1.53, 2.55)*	1.36 (1.01, 1.83)*

Values expressed as hazard ratio (95% confidence interval) (HR [95% CI]) and odds ratio (OR [95% CI]).

Adjusted for age, gender, diabetes mellitus, cardiovascular disease, current smoker, body mass index, mean arterial pressure, estimated glomerular filtration rate, log-transformed urine protein, albumin, hemoglobin, log-transformed C-reactive protein, glycated hemoglobin, uric acid, phosphate, statin and fibrate.

* *P*<0.05;

** *P*<0.001 compared to quintile 2.

### Total cholesterol quintiles and rapid renal progression

Odds ratios (OR) of the cholesterol quintiles for rapid renal progression are shown in Table 2. Either in unadjusted or adjusted models, quintile 5 with the highest total cholesterol was associated with increased risk for rapid renal progression and had faster renal function decline. The adjusted OR for quintile 5 *versus* quintile 2 was 1.36 (95% CI, 1.01 to 1.83, *P* = 0.043). The highest quintile of total cholesterol was independently associated with rapid renal progression.

### Other lipid profile and renal outcomes

Quintile 5 of other lipid profile (*versus* quintile 2) and RRT and rapid renal progression are shown in Table 3. In adjusted models for RRT, HR for LDL-cholesterol quintile 5 *versus* quintile 2 was 1.24 (95% CI, 1.02 to 1.51, *P* = 0.028) and HR for non-HDL-cholesterol quintile 5 *versus* quintile 2 was 1.28 (95% CI, 1.06 to 1.55, *P* = 0.010). The highest quintiles of LDL-cholesterol and non-HDL-cholesterol were independently associated with RRT. However, there was no significant correlation between LDL-cholesterol and non-HDL-cholesterol with rapid renal progression. As for triglyceride quintiles and HDL-cholesterol quintiles, there was no significant correlation between these two parameters with RRT and rapid renal progression.

**Table 3:** Quintile 5 of other lipid profile (*vs.* quintile 2) and renal replacement therapy and rapid renal progression in all subjects.

	Renal replacement therapy		Rapid renal progression	
	Unadjusted	Adjusted	Unadjusted	Adjusted
Quintile 5 of lipid profile	HR (95% CI)	HR (95% CI)	OR (95% CI)	OR (95% CI)
Quintile 5 of HDL-cholesterol (≥52 *vs.* quintile 2 : 32–36 mg/dL)	1.03 (0.85–1.25)	1.16 (0.95–1.41)	1.11 (0.86–1.44)	1.14 (0.84–1.53)
Quintile 5 of LDL-cholesterol (≥140 *vs.* quintile 2 : 83–100 mg/dL)	1.18 (0.97–1.42)	1.24 (1.02–1.51)*	1.73 (1.34–2.23)**	1.22 (0.91–1.64)
Quintile 5 of non-HDL-cholesterol (≥186 *vs.* quintile 2 : 114–136 mg/dL)	1.25 (1.04–1.50)*	1.28 (1.06–1.55)*	1.46 (1.14–1.86)*	1.16 (0.88–1.54)
Quintile 5 of triglyceride (≥202 *vs.* quintile 2 : 83–111 mg/dL)	1.03 (0.86–1.25)	1.10 (0.90–1.35)	1.30 (1.01–1.68)*	1.25 (0.93–1.67)

Values expressed as hazard ratio (95% confidence interval) (HR [95% CI]) and odds ratio (OR [95% CI]).

Adjusted for age, gender, diabetes mellitus, cardiovascular disease, current smoker, body mass index, mean arterial pressure, estimated glomerular filtration rate, log-transformed urine protein, albumin, hemoglobin, log-transformed C-reactive protein, glycated hemoglobin, uric acid, phosphate, statin and fibrate.

* *P*<0.05;

** *P*<0.001 compared to quintile 2.

## Discussion

In the present study, we evaluated the association of dyslipidemia and renal outcomes in patients with CKD stages 3–5. We found that either lower or higher total cholesterol, higher LDL-cholesterol, and higher non-HDL cholesterol were risk factors for RRT in stage 3–5 CKD patients. Higher total cholesterol was also significantly associated with rapid renal progression.

There is growing evidence that abnormalities in lipid metabolism contribute to renal disease progression [10], [11]. Our study also identified dyslipidemia as a risk factor for adverse renal outcome in stages 3–5 CKD. In patients with CKD, the abnormal lipoprotein metabolism results in dyslipidemia, including hypertriglyceridemia, increased triglyceride-rich lipoprotein remnants, reduced HDL-cholesterol, and increased lipoprotein (a) [1], [16]. The pathophysiological basis linking dyslipidemia and CKD is not only the aggravation of atherosclerosis in the renal microcirculation, but also deposition of lipoprotein in glomerular structures, and stimulates cytokines and growth factors involved in inflammation and fibrogenesis [17], [18]. Animal studies have shown that higher total cholesterol accelerates the rate of progression of kidney disease and. high-cholesterol feeding leads to macrophage infiltration and foam cells formation in rats [6], [18]. Results in human studies have not reached such an undisputed conclusion as in cellular and animal studies. In the Physician Health Study involving 4483 healthy males with an initial creatinine <1.5 mg/dl and a follow-up of 14.2 years, higher total cholesterol, higher non-HDL-cholesterol and lower HDL-cholesterol increased risk of developing renal dysfunction [10]. Muntner et al. studied the relationship of plasma lipids to a rise in serum creatinine of 0.4 mg/dL or greater in 12728 participants with baseline serum creatinine that was less than 2.0 mg/dL in men and less than 1.8 mg/dL in women. They found that individuals with higher baseline triglyceride and lower HDL-cholesterol levels were at increased risk for a rise in creatinine [19]. However, Chawala et al. investigated the relationship between dyslipidemia and renal outcomes in 840 non-diabetic CKS stage 3–4 patients. They used tertiles of lipid profiles (which might not reveal the U-shape relationship), and did not find significant correlation between dyslipidemia and renal outcomes [12]. Our study evaluated a CKD stages 3–5 cohort including diabetic and non-diabetic patients and verified that higher total cholesterol, higher LDL-cholesterol and higher non-HDL cholesterol impacted on renal function progression and an adverse renal outcome.

Another important finding of this study is that lower total cholesterol also increased risk for RRT. Several observational studies have demonstrated an association between lower total cholesterol and higher mortality in CKD and end-stage renal disease patients, and this seemingly paradoxical relationship may be explained by the high prevalence of malnutrition-inflammation [5], [20]. Iseki et al. showed lower total cholesterol was an independent predictor of death in patients on chronic hemodialysis. Impact of higher total cholesterol on survival was only evident in a subgroup of patients with serum albumin level higher than 4.5 g/dL [21]. These data suggested that low total cholesterol might actually represent a surrogate marker of malnutrition and inflammation. Recently, a concept of reverse epidemiology has been raised, which challenged the decisive roles of various conventional cardiovascular risk factors and the necessity of pharmaceutical management in renal failure patients [22], [23]. Instead, malnutrition and inflammation were recognized to be more important in this regard and tended to surpass these conventional factors. Malnutrition may worsen patients' outcomes by aggravating the existing inflammation and by accelerating atherosclerosis [23]–[26]. Our results showed that the lowest quintile of total cholesterol (*versus* quintile 2), which was associated with high prevalence of albumin <3.5 g/dL (28.1% *versus* 22.0%) and high CRP, was independently associated with progression to RRT. This implied that patients with malnutrition and inflammation, indexed by low total cholesterol level, might have rapid renal function decline and adverse renal outcome.

The idea that statins may slow renal disease progression has been of interest for nephrology practitioners. Clinical studies with statins on renal disease progression in patients with mild to moderate kidney failure have yielded conflicting results. The majority of these data come from post-hoc analyses or from randomized trials focused primarily on cardiovascular endpoints. Some of them suggest that statins slow the rate of renal function decline [27]–[29]. A meta-analysis by Sandhu et al. [29], including 27 randomized trials, examined the effects of statins therapy on kidney function in 39704 CKD stage 2–3 participants. When compared with placebo, statins therapy reduced the rate of renal function decline [29]. Other studies, however, have shown no benefits [30], [31]. These studies have several limitations, such as the presence of selection bias, short follow-up period, and lacking untreated CKD patients as control groups. More recently, the Study of Heart and Renal Protection (SHARP) [13] enrolling 9270 non-dialysis CKD patients with mild to moderate kidney failure examined the renal effect of lowering LDL-cholesterol with simvastatin plus ezetimibe. The main renal outcomes were end-stage renal disease, dialysis or transplantation. After a median follow-up of 4.9 years, there was no substantial effect on kidney disease progression despite substantial reduction in LDL-cholesterol levels [13]. However, SHARP study had quite low LDL-cholesterol (2.77 mmol/L) and ours had quite high LDL-cholesterol (2.94 mmol/L). Statins could still be beneficial in high LDL-cholesterol and total cholesterol patients. The condition of treatment (used or not used) with statins or fibrates was collected at the beginning, but the records about duration or dosage were lacking. Therefore, in our study, we were unable to evaluate the influence of statins or fibrates therapy on cholesterol and/or renal outcomes. Further study would be needed to determine whether lipid-lowering agents were helpful in improving renal outcomes. The available data and evidence to date are insufficient to conclude whether statins have an influence in slowing kidney disease progression in CKD patients.

In conclusion, our study in patients of CKD stage 3–5 showed dyslipidemia, either lower or higher total cholesterol, higher LDL-cholesterol, and higher non-HDL cholesterol were independently associated with RRT and rapid renal progression. Assessment of lipid profile may help identify high risk groups with adverse renal outcomes in CKD stage 3–5 patients.
